# The Immunostimulatory Capacity of Nontypeable *Haemophilus influenzae* Lipooligosaccharide

**DOI:** 10.20411/pai.v2i1.162

**Published:** 2017-02-16

**Authors:** Gabrielle N. Gaultier, Kayla N. Colledanchise, Alaa Alhazmi, Marina Ulanova

**Affiliations:** 1 Department of Biology, Lakehead University, Thunder Bay, ON, Canada; 2 Northern Ontario School of Medicine, Thunder Bay, ON, Canada

**Keywords:** Nontypeable *Haemophilus influenzae*, Lipooligosaccharide, THP-1 cells, innate immune responses

## Abstract

**Background:**

We have recently found that lipooligosaccharide (LOS) isolated from encapsulated strains of *Haemophilus influenzae* (*H. influenzae*) has strong adjuvant, but diminished pro-inflammatory ability as compared to *Escherichia coli* lipopolysaccharide (LPS). In this study, we aimed to determine the immunostimulatory capacity of nontypeable/ non-encapsulated *H. influenzae* (NTHi) LOS by comparing the effect of killed bacteria with LOS isolated from the same strain.

**Methods:**

Following stimulation of human monocytic THP-1 cells with killed NTHi strain 375, or with the corresponding amount of LOS, we studied the protein and gene expression of immunostimulatory and antigen-presenting molecules, cytokines, and innate immune receptors.

**Results:**

Stimulation with LOS resulted in lower expression of adhesion (CD54, CD58) as well as costimulatory molecules (CD40, CD86), but in higher expression of antigen-presenting molecules (HLA-DR and HLA-ABC) compared to killed NTHi, whereas killed bacteria induced higher release of both TNF-α and IL-10. The results indicate that while LOS of NTHi has decreased capacity to induce pro-inflammatory responses compared to *E. coli* LPS or killed NTHi, this LOS has the potential to facilitate antigen presentation.

**Conclusions:**

Considering the important role of NTHi as a respiratory pathogen, and its currently increasing significance in the etiology of invasive infections, LOS deserves further attention as a vaccine antigen, which also has potent adjuvant properties.

## INTRODUCTION

Nonencapsulated, or nontypeable *Haemophilus influenzae* (NTHi) is an important gram-negative human pathogen, which frequently colonizes the upper respiratory tract and is a common cause of local infections, such as sinusitis, pediatric otitis media, or exacerbations of chronic obstructive pulmonary disease (COPD) [1, 2]. During the last decade, an increasing incidence of invasive diseases caused by NTHi, including sepsis, meningitis, or bacteremic pneumonia has been reported worldwide, especially in newborns, immunocompromised individuals, and the elderly [3–5]. No specific vaccine to prevent NTHi infections is currently available, although a common *H. influenzae* antigen, protein D is included in the pediatric vaccine Synflorix^®^ as a carrier protein for pneumococcal polysaccharide antigens. Clinical trials demonstrated that this vaccine was 35% efficient in preventing otitis media caused by NTHi in young children; however, it remains unknown if Synflorix^®^ can offer protection against invasive NTHi disease [6]. Several *H. influenzae* antigenic compounds have been considered as potential NTHi-vaccine candidates, and some have been tested in animal models; however, none went beyond phase I clinical trials [7–12].

Lipooligosaccharide (LOS) is a major virulence factor of *H. influenzae* that is responsible for inflammatory responses associated with this infection [13]. This LOS is similar to one of the most studied endotoxin molecules, the lipopolysaccharide (LPS) of gram-negative bacteria. The structure of LPS consists of 3 covalently -linked components: lipid A embedded into the outer membrane, the oligosaccharide core, and the polysaccharide or O-antigen that covers the surface of the bacteria [14], and LPS activates the innate immune response once bound to the cellular receptor CD14, myeloid differentiation protein 2 (MD-2), and Toll-like receptor 4 (TLR4) [15]. Lipid A is the major pathogen-associated molecular pattern recognized by the innate immune system; the number of acyl chains determines the level of response that occurs [15]. In an *in vivo* situation, LPS causes a strong activation of the innate immune system that results in a massive production of pro-inflammatory cytokines clinically associated with the development of septic shock [14]. *Haemophilus influenzae* LOS has a similar lipid A structure and function to LPS, but it lacks repeating units of O-antigen side chain. The structure of LOS consists of an oligosaccha-ride core, which is made up of a triheptose that is linked to a 3-deoxy-D-manno-oct-2-ulosonic acid (Kdo) residue through covalent bonds. The core region is also covalently linked to the lipid A component, as well as an acylated glucosamine disaccharide backbone that is anchored to the outer membrane of bacteria [16, 17].

Our recent studies demonstrated that LOS isolated from either an encapsulated *H. influenzae* serotype b strain (Eagan), or a capsule-deficient serotype d strain (Rd) had diminished capacity to induce pro-inflammatory responses in comparison to a prototypic endotoxin of gram-negative bacteria, *Escherichia coli* LPS. However, LOS and LPS had comparable abilities to up-regulate the expression of co-stimulatory and antigen-presenting molecules [18]. Moreover, we have found the presence of naturally acquired bactericidal antibodies against NTHi LOS in sera of healthy adults suggesting their protective role against invasive disease [19]. Taking into consideration that LOS represents a common component of all *H. influenzae* strains and combines antigenic and adjuvant properties, it deserves further attention as a plausible vaccine candidate to protect against NTHi infections. In this study, we sought to elucidate the role of LOS in the overall immunostimulatory capacities of NTHi by comparing the effect of killed NTHi bacteria with the effect of LOS isolated from the same strain.

## MATERIALS AND METHODS

### Cell culture conditions

The human THP-1 monocytic leukemia cell line (ATCC, TIB-202) was stored in liquid nitrogen until thawed for culturing and maintained as described by Choi *et al* 2014 [18]. Cell number and viability were determined with a Bright-Line Hemacytometer (Hausser Scientific, Horsham, PA) using a 1:1 dilution factor with 0.4% Trypan blue solution (Sigma- Aldrich, St. Louis, MO). To induce differentiation, THP-1 cells were plated at 1 × 10^6^ cells/1 mL/well in 24-well flat-bottom plates (Costar, Corning Incorporated, Corning NY), in RPMI 1640 medium (Sigma-Aldrich) supplemented with 10% heat-inactivated fetal bovine serum (SAFC Biosciences, Lenexa, KS) and 200 μl antibiotic-antimycotic (complete medium) (Life Technologies, Inc., Burlington, Ontario). Cells were then treated with 20 ng/mL phorbol myristate acetate (PMA; Sigma-Aldrich) at 37°C in 5% CO_2_ for 12 hours, then washed and re-suspended in the same medium. After 48 hours of further incubation, the cells were washed twice with serum- and antibiotic-free medium and used for experiments.

### Bacterial culture

NTHi strain 375, originally isolated from a pediatric patient with otitis media [20] was grown on brain-heart infusion (BHI) plates supplemented with 10 mg/mL hemin and 1 μg/mL nicotine adenine dinucleotide (NAD) overnight, in 5% CO_2_ at 37°C. Bacteria were then transferred to supplemented BHI broth and grown to log phase at 37°C with shaking. To kill bacteria, a NTHi suspension with an optical density (OD) of 0.1 read at 600 nm was prepared and treated with 100 μg/mL gentamicin (Sigma-Aldrich) for 30 minutes at 37°C with shaking. Complete killing was confirmed using drop plating. Bacteria were washed twice with phosphate buffered saline (PBS), resuspended in PBS, and used for stimulation of THP-1 cells. The LOS from NTHi 375 was isolated and purified using proteinase K as described by Choi *et al* [18].

### Estimation of the number of bacteria harboring certain amounts of LOS

In order to estimate the number of bacteria that contain certain amounts of LOS, previously reported values of cell-bound LOS were used. Gu *et al* determined the range of cell-bound LOS per bacterium for NTHi strains 9274, 6491, 5756, 2627, and 2019 as 1.6 × 10^6^ to 4.8 × 10^6^ molecules [21]. For our calculations, we considered an average of the lowest and highest reported values, ie, 3.2 × 10^6^ molecules of LOS per bacterium. Using this value and Avogadro's number (6.022 × 10^23^), the moles of LOS per bacterium were determined. As the molecular weight of LOS has been estimated as 4,000 g/mol, based on known structures of oligosaccharides and lipid A from *H. influenzae* LOS [21], it was multiplied by the moles of LOS per bacterium to obtain an average weight of LOS per bacterium and used to determine the number of bacteria harboring certain amounts of LOS as indicated in Table 1. The numbers of bacteria used for experiments were divided by the number of THP-1 cells to determine the approximate NTHi to THP-1 cell ratio (Table 1).

**Table 1.** Estimated numbers of bacteria corresponding to LOS doses used in this study.

LOS (μg/mL)	Number of NTHi 375 (CFU)	Number of THP-1 cells	NTHi/THP-1 ratio
0.1	4.762 × 10^6^	1.5 × 10^6^	6*
1.0	4.762 × 10^7^	1.5 × 10^6^	60*
1.0	4.762 × 10^7^	0.5 × 10^6^	190**
5.0	2.381 × 10^8^	0.5 × 10^6^	952**
10.0	4.762 × 10^8^	0.5 × 10^6^	1905**
15.0	7.143 × 10^8^	0.5 × 10^6^	2857**

* Gene expression analysis (qPCR),

** Surface molecule expression (flow cytometry and ELISA analysis)

### Flow cytometry analysis

A total of 0.5 × 10^6^ THP-1 cells in 2 mL of complete medium were placed in each well of a 12-well plate (Costar) for 24 hours. Cells were stimulated with 1 μg/mL of *E. coli* 0111:B4 LPS (Sigma-Aldrich), or 1, 5, 10, or 15 μg/mL NTHi 375 LOS, or the corresponding amounts of killed bacteria for 24 hours as described by Choi *et al* [18]. Following stimulation, cells were washed once with PBS and immunostained with fluorochrome- conjugated antibodies against CD54 (ICAM-1), CD40, CD86 (B7-2) (BD Biosciences, Mississauga, Ontario), CD58 (LFA-3) (Cedarlane, Burlington, Ontario), HLA-DR (major histocompatibility complex (MHC) class II) (Biolegend, San Diego, CA) HLA-ABC (MHC class I) antibody (BD Biosciences) or the corresponding isotype control as was previously described [18].

### Gene expression analysis

The experiments were performed as previously described [18]. Briefly, 1.5 × 10^6^ THP-1 cells were suspended in 2 mL complete medium and the concentrations of 0.1 and 1 μg/mL of NTHi 375 LOS, or *E. coli* LPS were used to stimulate the cells for 4 hours, followed by RNA extraction and qPCR analysis. Gene expression of TNF-α, IL-10, IL-1β, TLR4, and NOD2 was measured and normalized to the housekeeping gene Peptidylprolyl isomerase B (PPIB) (SA Biosciences, Mississauga, Ontario) to determine the fold change for each gene of interest [18].

## ELISA

Differentiated THP-1 cells were stimulated with NTHi LOS at concentrations of 1, 5, 10, and 15 μg/ ml, or corresponding amounts of killed bacteria, or *E. coli* 0111:B4 LPS at concentration of 1 μg/mL for 24 hours. Cell culture supernatants were aliquoted and stored at −80°C for cytokine measurement. The concentrations of IL-1β, TNF-α, IL-10, and INF-γ were determined by ELISA according to the manufacturer's instructions (eBioscience, San Diego, CA). The detection limits of the assays were 2 pg/mL for IL-1β and IL-10, and 4 pg/mL for TNF-α and INF-γ.

## STATISTICAL ANALYSIS

Data were expressed as a mean of at least 3 independent experiments. Statistical significance was determined with a 1-way analysis of variance (ANOVA) with Newman-Keuls multiple comparison post hoc test, where *P* values less than 0.05 were considered significant (Prism, GraphPad version 5.0, La Jolla, CA).

## RESULTS

### LOS isolated from NTHi has decreased pro-inflammatory, but similar adjuvant ability to the prototypic LPS

**Figure 1.**
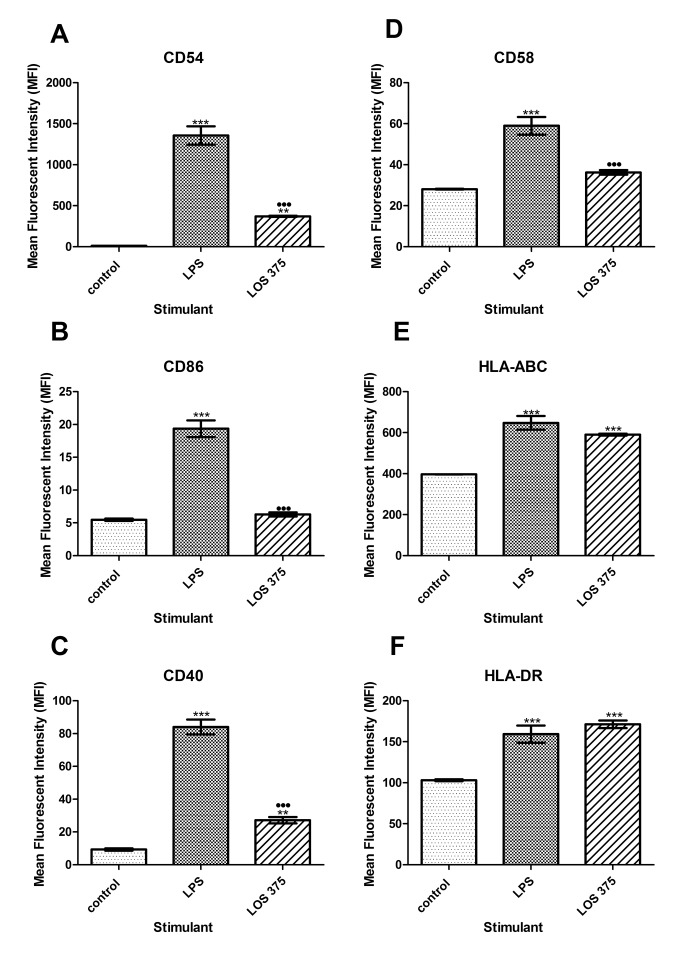
Flow cytometry analysis of the expression of co-stimulatory and antigen-presenting molecules in THP-1 cells stimulated with NTHi LOS or *E. coli* LPS. THP-1 cells were stimulated with 1.0 μg/ml of LPS or LOS for 24 hours. Unstimulated THP-1 cells (0.5 × 10^6^) in complete culture medium served as a negative control. Cell surface expression of CD54 (A), CD86 (B), CD40 (C), CD58 (D) HLA-ABC (E), and HLADR (F) was measured and data expressed as the mean fluorescence intensity (MFI); the bars indicate the mean with the standard error of the mean (SEM) (n = 3). *, *P* < 0.05 compared to the negative control; ●, *P* < 0.05 between LPS and LOS.

To study the ability of LOS to activate innate immune responses, THP-1 cells were stimulated with either NTHi 375 LOS, or a potent innate immune activator *E. coli* 0111:B4 LPS, and cell surface expression of CD54 (ICAM-1), CD40, CD58 (LFA-1), CD86 (B7), HLA- ABC (MHC class I), and HLA-DR (MHC class II) was assessed using flow cytometry analysis. As a negative control, unstimulated THP-1 cells were used. Although stimulation with NTHi LOS caused upregulation of CD54, CD40, and CD58 expression in THP-1 cells, this effect was significantly reduced compared to stimulation with the same dose of *E. coli* LPS, ie 1 μg/mL (Figure 1A, C, D). However, NTHi LOS and *E. coli* LPS induced a similar increase in the expression of both MHC class I and II molecules (Figure 1E, F). Unlike the effect of LPS, stimulation of THP-1 cells with 1 μg/mL of NTHi LOS did not cause an increase in the expression of CD86 (Figure 1B).

**Figure 2.**
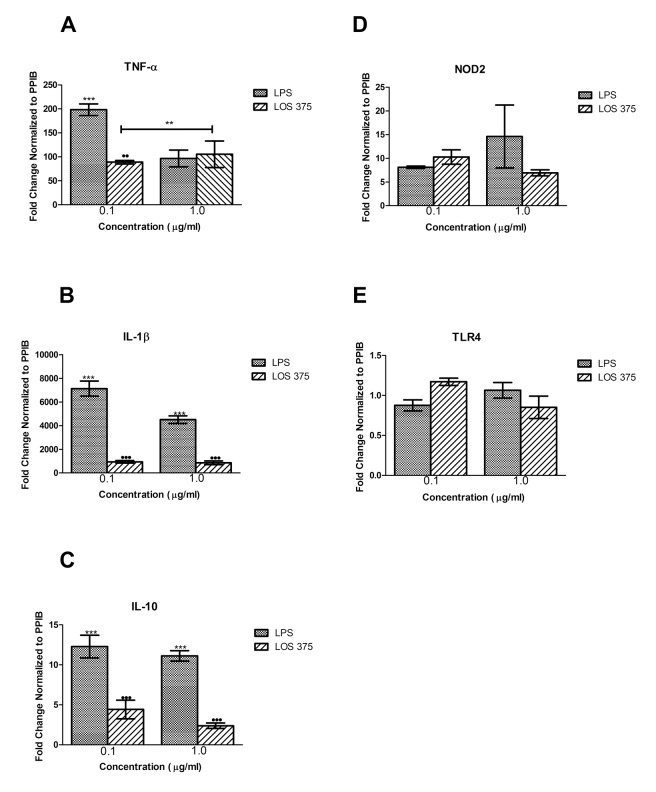
Gene expression analysis for THP-1 cells stimulated with NTHi LOS or *E. coli* LPS. THP-1 cells were stimulated with either LPS or LOS at 0.1 and 1 μg/ml for 4 hours. Following RNA extraction, gene expression of TNF-α (A), IL-1β (B), IL-10 (C), NOD2 (D), and TLR4 (E) was studied using real-time PCR. Data are displayed as the fold change relative to the unstimulated control. Data represent means ± SEM (n = 3). *, *P* < 0.05 compared to unstimulated control; ●, *P* < 0.05 between LPS and LOS.

These results indicate that although LOS of NTHi 375 shows decreased ability to induce the surface expression of co-stimulatory molecules CD54, CD40, CD58, and CD86 as compared to the same dose of LPS, it has a similar ability to up-regulate the expression of antigen-presenting molecules MHC class I and II. Furthermore, LOS has decreased ability to induce gene expression of pro-inflammatory cytokines TNF-α and IL-1β as well as of an anti-inflammatory cytokine IL-10 compared to LPS, at concentrations of both 0.1 and 1 μg/mL (Figure 2). Interestingly, LOS and LPS had a comparable ability to up-regulate the gene expression of an intracellular pattern-recognition receptor NOD2, although neither one had an effect on TLR4 gene expression (Figure 2).

### LOS isolated from NTHi has decreased pro-inflammatory ability in comparison to whole bacterial cells

**Figure 3.**
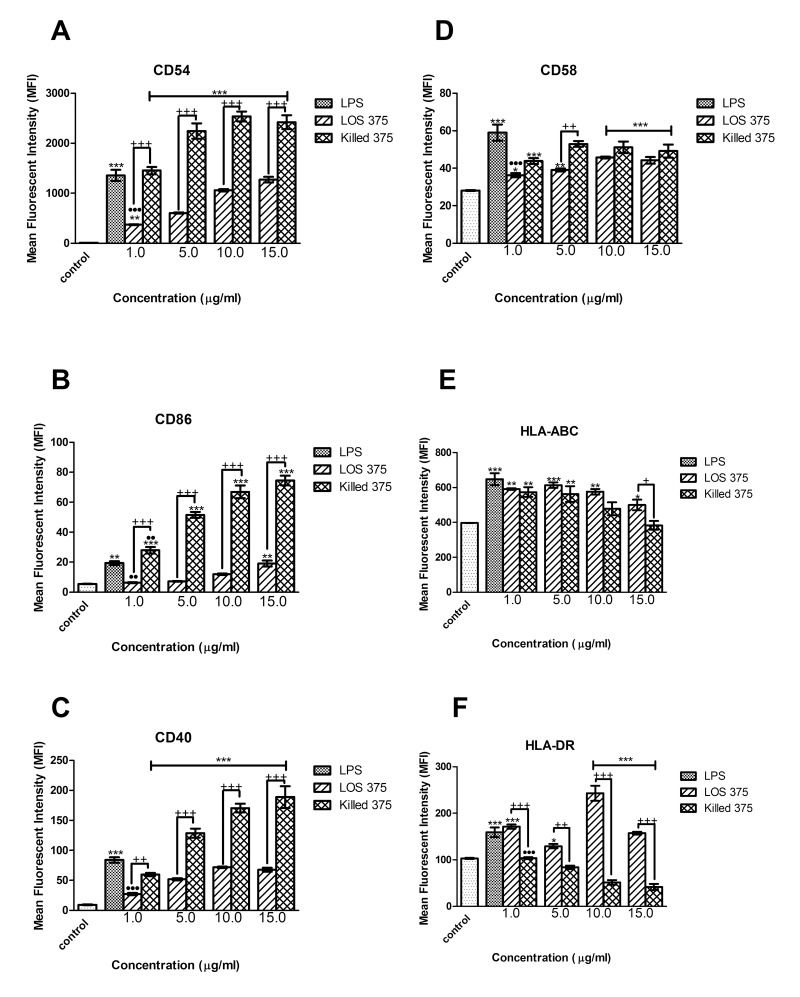

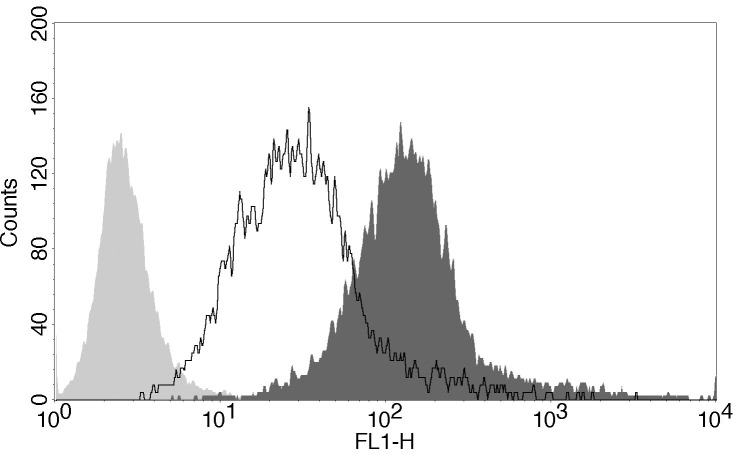
Flow cytometry analysis of the expression of co-stimulatory and antigen-presenting molecules in THP-1 cells stimulated with *E. coli* LPS, NTHi LOS or killed NTHi. THP-1 cells were stimulated with 1.0 μg/ml of LPS, or LOS at concentrations of 1, 5, 10, and 15 μg/ml, or corresponding amounts of killed NTHi: 4.762 × 10^7^, 2.381 × 10^8^, 4.762 × 10^8^, 7.143 × 10^8^ CFU per 0.5 × 10^6^ THP-1 cells for 24 hours. Un-stimulated THP-1 cells (0.5 × 10^6^) in complete culture medium served as a negative control. Cell surface expression of CD54 (A), CD86 (B), CD40 (C), CD58 (D) HLA-ABC (E), and HLA-DR (F) was measured and data expressed as the mean fluorescence intensity (MFI) with the standard error of the mean (SEM) (n = 3). *, *P* < 0.05 compared to the unstimulated control; +, *P* < 0.05 between LOS and killed NTHi; ●, *P* < 0.05 between LPS and LOS. (G): Original histograms of 1 representative experiment showing HLA-DR expression in THP-1 cells stimulated with 10 μg/ml of NTHi LOS (dark grey), or with the corresponding amounts of killed NTHi (open histogram); light grey: unstimulated cells stained with the isotype control antibody.

To examine the contribution of NTHi LOS to the immunostimulatory ability of whole bacteria we compared the results of the exposure of THP-1 cells to increasing doses of LOS with the corresponding numbers of killed NTHi of the same strain. In comparison to the effect of killed NTHi, LOS stimulation resulted in lower expression of CD54, CD40, and CD86 (at doses between 1 and 15 μg/ mL), and of CD58 (at doses of 1 and 5 μg/mL) (Figure 3A-D) suggesting that in addition to LOS, other immunostimulatory bacterial components may contribute to the upregulation of these surface molecules. The effect of LOS was dose-dependent, and in the case of CD86, the highest LOS dose, (15 μg/mL), induced the expression of this co-stimulatory molecule in a manner comparable to LPS (Figure 3B).

Interestingly, LOS induced significantly higher expression of antigen-presenting molecules compared to the effect of the corresponding numbers of whole bacteria. For HLA-DR, this effect was consistent for all the doses of LOS; for HLA-ABC, the effect only appeared at the highest LPS concentration (15 μg/mL) (Figure 3E-G). Because the cellular expression of MHC molecules is largely regulated by a balance of the cytokines IFN-γ and IL-10, we studied their release by differentiated THP-1 cells stimulated with purified LOS or killed NTHi.

### Effect of LOS on cytokine release in comparison to whole bacterial cells

**Figure 4.**
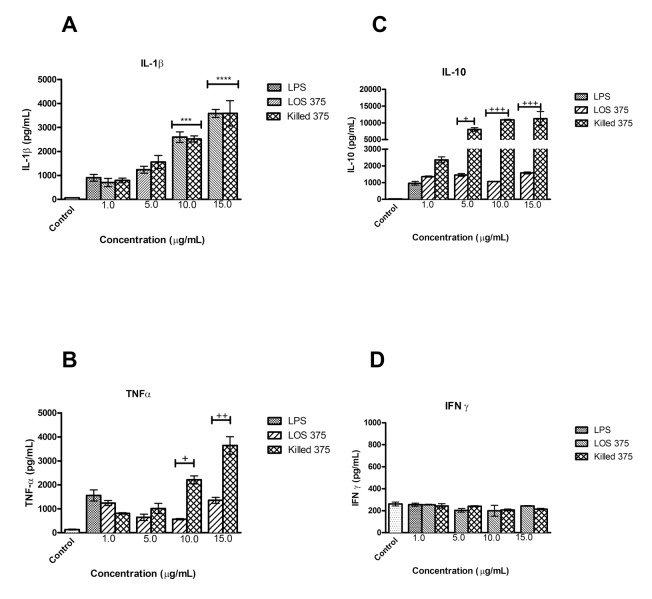
Cytokine release from differentiated THP-1 cells stimulated with NTHi LOS or killed NTHi for 24 hours. THP-1 cells were stimulated with LOS at concentrations of 1, 5, 10, and 15 μg/ml, or corresponding amounts of killed NTHi 375: 4.762 × 10^7^, 2.381 × 10^8^, 4.762 × 10^8^, 7.143 × 10^8^ CFU per 1 × 10^6^ cells. Unstimulated differentiated THP-1 cells (1 × 10^6^) in complete culture medium served as a negative control; cells stimulated with 1 μg/ml of *E. coli* LPS served as a positive control. Concentrations of IL-1β (A), TNFα (B), IL-10 (C), and IFNγ (D) were measured by ELISA. Data represents mean cytokine concentration ± SEM (n = 3). *, *P* < 0.05 compared to LPS; +, *P* < 0.05 between LOS and killed NTHi.

To study the release of cytokines TNF-α, IL-1β, IL-10, and IFN-γ, which are important in innate immune responses to bacterial pathogens, we stimulated PMA-differentiated THP-1 cells that acquired certain characteristics of macrophages with increasing doses of LOS, or the corresponding numbers of killed NTHi. Our preliminary experiments indicated that un-differentiated THP-1 cells had low cytokine production capacity (data not shown). Stimulation of differentiated THP-1 cells with either LOS or killed NTHi induced a dose-dependent increase in the levels of IL-1β, TNF-α, and IL-10 released by THP-1 cells that was comparable to the effect of LPS when used at the same concentration 1 μg/mL (Figure 4A-C). However, IFN-γ was not induced by any treatment (Figure 4D). Although there was no difference between the effect of LOS and whole bacteria with regard to the release of IL-1β, whole NTHi induced a significantly higher release of TNF-α and IL-10 in comparison to LOS (for TNF-α, it was apparent at LOS concentrations of 10 and 15 μg/mL; for IL-10, at LOS concentrations of 5, 10, and 15 μg/mL).

## DISCUSSION

Clinical infections caused by NTHi are characterized by intense inflammation that has been largely attributed to LOS, the major component of the *H. influenzae* cell wall [22]; LOS has the capacity to activate pro-inflammatory responses due to the interaction of lipid A with TLR4-signaling complex resulting in NF-κB activation with subsequent expression of multiple pro-inflammatory molecules [23]. Indeed, in our model, we observed that exposure of human monocytic cells to killed NTHi *in vitro* resulted in a large release of TNF-α, a hallmark of NF-κB activation [24]. However, when we compared the effect of LOS isolated from the same NTHi strain with the effect of whole bacteria containing the corresponding amounts of LOS, it became obvious that LOS was only partially responsible for the pro-inflammatory capacity of NTHi. This is not surprising, as multiple bacterial compounds have been demonstrated to activate the innate immune system and induce inflammatory responses. In particular, *H. influenzae* peptidoglycan is able to induce inflammatory responses following its recognition by the intracellular receptor NOD2 [25]. Interestingly, we found that NTHi LOS caused upregulation of NOD2 gene expression that was comparable to the effect of *E. coli* LPS suggesting that NOD2 could also be involved in the recognition of LOS and LPS [26–28].

Other NTHi molecules that can potentially be recognized by innate immune receptors and are capable of inducing inflammatory responses include lipopeptides and porins (TLR2 ligands), and bacterial DNA (TLR9 ligand) [29–33]. Moreover, a cross talk among different pattern recognition receptors activated by several microbial products can result in the amplification of cellular responses [23]. Recent studies indicated that NTHi could activate the NLRP3 inflammasome [34]. Indeed, we found that both whole NTHi and isolated LOS induced release of mature IL-1β that requires inflammasome activation, in addition to the transcription of the IL-1β gene, which is largely regulated by NF-κB [35].

The results of this study corroborate our earlier observations that the pro-inflammatory ability of *H. influenzae* LOS is significantly decreased in comparison to *E. coli* LPS, the prototypic endotoxin of gram-negative bacteria [18]. In particular, the ability of LOS isolated from *H. influenzae* serotype b strain Eagan and from the serotype d strain Rd to induce expression of pro-inflamma-tory molecules was diminished in comparison to that of the LPS [18]. The present study expands our previous observations to LOS from an NTHi strain that was isolated from a pediatric case of otitis media [20] indicating that the capacity of *H. influenzae* LOS to activate innate immune cells is not restricted to particular serotypes of the pathogen.

It is well established that NTHi bacteria are distinct and more genetically variable compared to encapsulated *H. influenzae*, and considerable heterogeneity exists among the structures of lipid A expressed by different *H. influenzae* strains [36, 37]. Because such heterogeneity may be manifested in differentially acylated lipid A forms that would determine the interaction with the TLR4 receptor complex, it may potentially result in different degrees of immunostimulatory ability among the different strains. It is interesting that despite the great genetic diversity among *H. influenzae* strains, LOS of NTHi shares immunostimulatory capacity with LOS isolated from Eagan and Rd [18].

In fact, LOS of NTHi strain 375, similarly to LOS of Eagan and Rd, had a marked ability to induce the expression of both MHC class I and II antigen-presenting molecules that was similar to those of *E. coli* LPS. Moreover, we found that NTHi LOS induced higher expression of HLA-DR as compared to the corresponding numbers of killed NTHi. This may be explained by different effects of whole bacteria versus isolated LOS on the production of the anti-inflammatory cytokine IL-10. Differentiated THP-1 cells that acquired macrophage-like properties produced lower amounts of IL-10 when stimulated with LOS as compared to the corresponding numbers of whole bacteria. This is not surprising because production of IL-10 is regulated by signaling initiated by the engagement of several pattern-recognition receptors including TLR2, TLR4, TLR9, and Nod-like receptors [38]. Because IL-10 is known to down-regulate the cellular expression of MHC molecules [39], its higher production induced by whole NTHi may explain differences in MHC class II expression induced by LOS versus NTHi. Of note, IFN-γ which is the major cytokine up-regulating the MHC class II expression on monocytes [40] was not induced by either whole killed NTHi or LOS. Indeed, production of IFN-γ by human macrophages could require additional signals, such as IL-12 in combination with IL-18 [41]. Although using killed bacteria in an experimental model does not reproduce the full spectrum of biological events triggered by NTHi *in vivo*, our approach can nevertheless be clinically relevant. In cases of NTHi infections treated with antibiotics, the immune system is exposed to large numbers of killed bacteria, and hence cellular responses studied in our model may reflect actual activation of innate immune cells, including both immature monocytes and macrophage-like differentiated monocytic cells.

The results of this study suggest that isolated LOS of NTHi may represent an attractive vaccine candidate to prevent invasive disease caused by this pathogen. Importantly, isolated LOS had decreased pro-inflammatory ability as compared to typical LPS of gram-negative bacteria or whole NTHi bacteria, specifically in terms of TNF-α and IL-1β production. However, LOS showed the capacity to upregulate the expression of co-stimulatory molecules CD54 (ICAM-1), CD58 (LFA-3), CD86 (B-7), and CD40, which are essential for T- and B-lymphocyte engagement. Moreover, LOS stimulated a high expression of antigen-presenting molecules, HLA-DR and HLA-ABC suggesting that LOS exerts an adjuvant effect facilitating the adaptive immune responses to the pathogen. We have previously detected the presence of LOS-specific IgG and IgM antibodies exhibiting bactericidal activity towards NTHi strain 375 in the sera of healthy adult individuals, which may potentially have a protective effect against systemic NTHi infections [19], and therefore the immunostimulatory effect of NTHi LOS found in the present study may have biological relevance. This study emphasizes the need for further attention to be given to LOS as a vaccine antigen, which also has adjuvant properties; this is of particular importance because of the increasing significance of NTHi as a cause of invasive disease in addition to its major role in local mucosal infections. However, more research involving primary cells as well as experimental animal models is required for further exploration of LOS as a potential vaccine candidate against NTHi.
